# Di­chlorido­(5,10,15,20-tetra­phenyl­porphyrinato-κ^4^ *N*)anti­mony(V) hexa­chlorido­anti­monate(V)

**DOI:** 10.1107/S1600536814012653

**Published:** 2014-06-11

**Authors:** Raoudha Soury, Mohamed Salah Belkhiria, Michel Giorgi, Habib Nasri

**Affiliations:** a Laboratoire de Physico-Chimie des Matériaux, Université de Monastir, Faculté des Sciences de Monastir, Avenue de l’environnement, 5019 Monastir, Tunisia; b Spectropole, Université d’Aix-Marseille, Faculté des Science, St-Jérôme, Avenue Escadrille Normandie-Niemen, 13397 Marseille Cedex 20, France

## Abstract

The asymmetric unit of the title compound, [Sb(C_44_H_28_N_4_)Cl_2_][SbCl_6_], consists of one half of an anti­mony(V) tetra­phenyl­porphyrin complex cation and one half of an hexa­chlorido­anti­monate(V) anion. In the complex cation, the Sb^V^ atom lies on an inversion center and is octa­hedrally coordinated by four N atoms from a macrocyclic tetra­phenyl­porphyrinate ligand and two chloride ions. The complex cation has approximately a planar core with a maximum deviation of 0.018 (5) Å from the porphyrin mean plane. The average Sb—N distance is 2.062 (11) Å, while the Sb—Cl distance is 2.355 (1) Å. The Sb^V^ atom of the anion is also located on an inversion center. The [SbCl_6_]^−^ octa­hedron exhibits rhombic distortion characterized by the Sb—Cl bond lengths [2.311 (3), 2.374 (2) and 2.393 (4) Å]. In the crystal, the cations and anions are linked C—H⋯ Cl hydrogen bonds, forming a layer parallel to (211).

## Related literature   

For general background and the synthesis, see: Liu *et al.* (1996 ▶). For related structures, see: Tsunami *et al.* (2008 ▶); Soury *et al.* (2012 ▶).
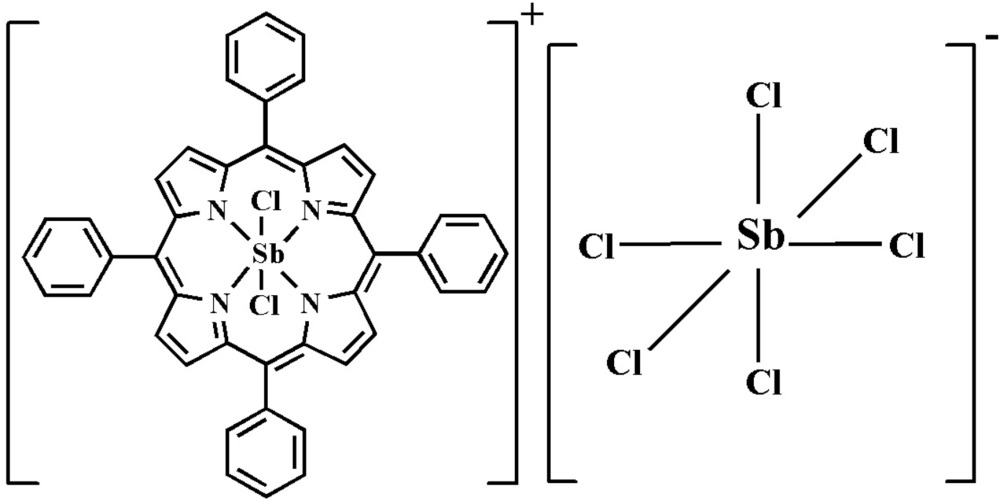


## Experimental   

### 

#### Crystal data  

[Sb(C_44_H_28_N_4_)Cl_2_][SbCl_6_]
*M* *_r_* = 1139.80
Triclinic, 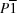

*a* = 10.2044 (4) Å
*b* = 11.1242 (4) Å
*c* = 11.3901 (4) Å
α = 70.685 (2)°
β = 83.398 (2)°
γ = 63.050 (3)°
*V* = 1086.73 (7) Å^3^
*Z* = 1
Mo *K*α radiation
μ = 1.77 mm^−1^
*T* = 223 K
0.2 × 0.16 × 0.12 mm

#### Data collection  

Bruker–Nonius KappaCCD diffractometer
Absorption correction: multi-scan (*SORTAV*; Blessing, 1995 ▶) *T*_min_ = 0.723, *T*_max_ = 0.773
19871 measured reflections
4749 independent reflections
4433 reflections with *I* > 2σ(*I*)
*R*_int_ = 0.124

#### Refinement  

*R*[*F* ^2^ > 2σ(*F* ^2^)] = 0.065
*wR*(*F* ^2^) = 0.173
*S* = 1.13
4749 reflections
266 parameters
H-atom parameters constrained
Δρ_max_ = 2.55 e Å^−3^
Δρ_min_ = −1.45 e Å^−3^

### 

Data collection: *COLLECT* (Nonius, 2002 ▶); cell refinement: *DENZO*/*SCALEPACK* (Otwinowski & Minor, 1997 ▶); data reduction: *DENZO*/*SCALEPACK*; program(s) used to solve structure: *SIR2004* (Burla *et al.*, 2005 ▶); program(s) used to refine structure: *SHELXL97* (Sheldrick, 2008 ▶); molecular graphics: *ORTEP-3 for Windows* (Farrugia, 2012 ▶); software used to prepare material for publication: *SHELXL97*.

## Supplementary Material

Crystal structure: contains datablock(s) I, New_Global_Publ_Block. DOI: 10.1107/S1600536814012653/is5356sup1.cif

Structure factors: contains datablock(s) I. DOI: 10.1107/S1600536814012653/is5356Isup2.hkl

CCDC reference: 1006075

Additional supporting information:  crystallographic information; 3D view; checkCIF report

## Figures and Tables

**Table 1:** Hydrogen-bond geometry (Å, °)

*D*—H⋯*A*	*D*—H	H⋯*A*	*D*⋯*A*	*D*—H⋯*A*
C7—H7⋯Cl3^i^	0.94	2.76	3.490 (8)	135
C8—H8⋯Cl4	0.94	2.74	3.593 (8)	151
C12—H12⋯Cl3^ii^	0.94	2.69	3.539 (8)	151

Symmetry codes: (i) 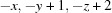; (ii) 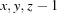.

**Fig. 1.**
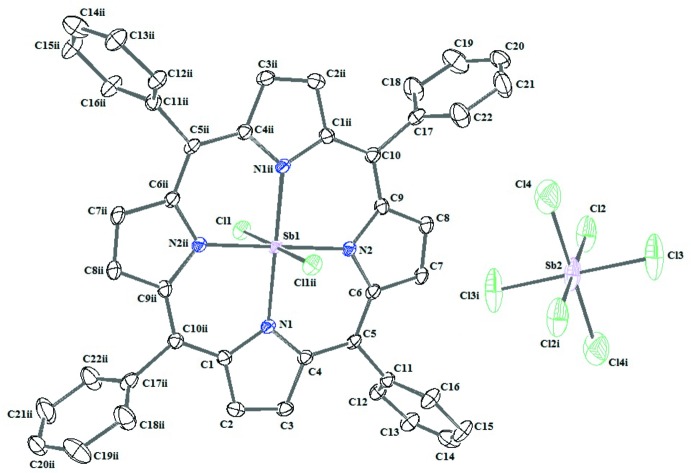
The molecular structure of the title compound with displacement ellipsoids drawn at 30% probability level. Hydrogen atoms have been omitted for clarity.

**Fig. 2.**
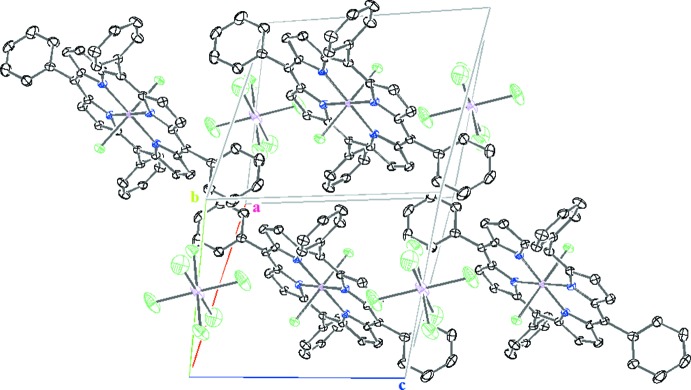
A crystal packing diagram of the title compound viewed along the b axis.

## supplementary crystallographic information

### S1. Comment

As part of our continuing studies of antimony porphyrin complexes (Soury *et al.*, 2012), we report here the synthesis and crystal structure of the tiltle compound [Sb(tpp)Cl_2_][SbCl_6_] which appears to be the eleventh X-ray structure of an antimony porphyrin complex in the literature.

The molecular structures of the antimony(V) porphyrin cation [Sb(tpp)Cl_2_]^+^ and the hexachloridoantimonate(V) [SbCl_6_]^-^ anion of the title compound are given in Figure 1. The Sb atom of the porphyrin complex lies on an inversion center and at the same time in the porphyrin mean plane (C_24_N_4_). The average Sb—N distance and the Sb—Cl distance values, respectively equal to 2.062 (4) Å and 2.355 (1) Å, agree very well with those reported in literature (Liu *et al.*, 1996; Tsunami *et al.*, 2008). The Sb atom of the counter-anion is located on an inversion center and has a rhombic distorted octahedral environment with three values of Sb—Cl bond lengths [2.311 (3), 2.374 (2) and 2.393 (4) Å]. Such bond distances are comparable to those observed for similar porphyrin complexes (Soury *et al.*, 2012). The cohesion of the structure is ensured by C—H··· Cl hydrogen bond interactions (Table 1). The crystal packing of compound [Sb(tpp)Cl_2_][SbCl_6_] is presented in Figure 2.

### S2. Experimental

The title compound [Sb(tpp)Cl_2_][SbCl_6_] synthesis was performed under argon atmosphere. SbCl_5_ (3–4 cm^3^) was added to a solution of tetraphenylporphyrin (H_2_tpp) (500 mg, 0.814 mmol) in pyridine (25 cm^3^) and the resulting mixture was refluxed for 1 h. After removing pyridine and excess antimony pentachloride by rotary evaporation, the purple solid obtained was dissolved in dichloromethane and chromatographed on silica gel 60 (100 g, neutral, activity I). The reaction mixture was firstly eluted with CH_2_C1_2_ to eliminate any free H_2_tpp present and then the title compound was eluted as a purple band using CH_2_C1_2_-methanol (2%). Single red crystals of the title complex, suitable for X-ray diffraction, have been obtained by diffusion of hexanes in dichloromethane solution.

### S3. Refinement

All H atoms were refined using a riding model with C—H = 0.93 Å and *U*_iso_(H) = 1.2*U*_eq_(C).

### Crystal data

**Table:**

[Sb(C_44_H_28_N_4_)Cl_2_][SbCl_6_]	*Z* = 1
*M**_r_* = 1139.80	*F*(000) = 558
Triclinic, *P*1	*D*_x_ = 1.742 Mg m^−^^3^
Hall symbol: -P 1	Mo *K*α radiation, λ = 0.71073 Å
*a* = 10.2044 (4) Å	Cell parameters from 22201 reflections
*b* = 11.1242 (4) Å	θ = 1.9–30.1°
*c* = 11.3901 (4) Å	µ = 1.77 mm^−^^1^
α = 70.685 (2)°	*T* = 223 K
β = 83.398 (2)°	Prism, red
γ = 63.050 (3)°	0.2 × 0.16 × 0.12 mm
*V* = 1086.73 (7) Å^3^	

### Data collection

**Table:**

Bruker–Nonius KappaCCD diffractometer	4749 independent reflections
Radiation source: fine-focus sealed tube	4433 reflections with *I* > 2σ(*I*)
Graphite monochromator	*R*_int_ = 0.124
φ & ω scans	θ_max_ = 27.1°, θ_min_ = 1.9°
Absorption correction: multi-scan (*SORTAV*; Blessing, 1995)	*h* = −13→13
*T*_min_ = 0.723, *T*_max_ = 0.773	*k* = −14→14
19871 measured reflections	*l* = −14→14

### Refinement

**Table:**

Refinement on *F*^2^	Secondary atom site location: difference Fourier map
Least-squares matrix: full	Hydrogen site location: inferred from neighbouring sites
*R*[*F*^2^ > 2σ(*F*^2^)] = 0.065	H-atom parameters constrained
*wR*(*F*^2^) = 0.173	*w* = 1/[σ^2^(*F*_o_^2^) + (0.0507*P*)^2^ + 7.0167*P*] where *P* = (*F*_o_^2^ + 2*F*_c_^2^)/3
*S* = 1.13	(Δ/σ)_max_ < 0.001
4749 reflections	Δρ_max_ = 2.55 e Å^−^^3^
266 parameters	Δρ_min_ = −1.45 e Å^−^^3^
0 restraints	Extinction correction: *SHELXL97* (Sheldrick, 2008), Fc^*^=kFc[1+0.001xFc^2^λ^3^/sin(2θ)]^-1/4^
Primary atom site location: structure-invariant direct methods	Extinction coefficient: 0.042 (4)

### Special details

**Table:**

Geometry. All s.u.'s (except the s.u. in the dihedral angle between two l.s. planes) are estimated using the full covariance matrix. The cell s.u.'s are taken into account individually in the estimation of s.u.'s in distances, angles and torsion angles; correlations between s.u.'s in cell parameters are only used when they are defined by crystal symmetry. An approximate (isotropic) treatment of cell s.u.'s is used for estimating s.u.'s involving l.s. planes.
Refinement. Refinement of *F*^2^ against ALL reflections. The weighted *R*-factor *wR* and goodness of fit *S* are based on *F*^2^, conventional *R*-factors *R* are based on *F*, with *F* set to zero for negative *F*^2^. The threshold expression of *F*^2^ > 2σ(*F*^2^) is used only for calculating *R*-factors(gt) *etc*. and is not relevant to the choice of reflections for refinement. *R*-factors based on *F*^2^ are statistically about twice as large as those based on *F*, and *R*- factors based on ALL data will be even larger.

### Fractional atomic coordinates and isotropic or equivalent isotropic displacement parameters (Å^2^)

**Table:**

	*x*	*y*	*z*	*U*_iso_*/*U*_eq_	
Sb1	0.5000	0.0000	0.5000	0.0226 (2)	
N1	0.4725 (5)	0.1913 (5)	0.3693 (4)	0.0259 (9)	
N2	0.3841 (5)	0.1056 (5)	0.6238 (4)	0.0261 (9)	
Cl1	0.28107 (16)	0.02557 (17)	0.42325 (15)	0.0368 (4)	
C1	0.5256 (6)	0.2093 (6)	0.2499 (5)	0.0278 (11)	
C2	0.4834 (7)	0.3574 (7)	0.1912 (6)	0.0358 (13)	
H2	0.5047	0.3994	0.1098	0.043*	
C3	0.4073 (7)	0.4267 (6)	0.2732 (5)	0.0317 (12)	
H3	0.3669	0.5251	0.2589	0.038*	
C4	0.3993 (6)	0.3234 (6)	0.3857 (5)	0.0270 (11)	
C5	0.3287 (6)	0.3531 (5)	0.4932 (5)	0.0265 (11)	
C6	0.3224 (6)	0.2509 (6)	0.6023 (5)	0.0273 (11)	
C7	0.2497 (6)	0.2797 (6)	0.7118 (6)	0.0312 (12)	
H7	0.1987	0.3696	0.7229	0.037*	
C8	0.2678 (7)	0.1537 (7)	0.7969 (6)	0.0341 (13)	
H8	0.2310	0.1416	0.8774	0.041*	
C9	0.3518 (7)	0.0429 (6)	0.7440 (5)	0.0292 (11)	
C10	0.3941 (6)	−0.1013 (6)	0.8037 (5)	0.0286 (11)	
C11	0.2526 (6)	0.5050 (6)	0.4938 (5)	0.0287 (11)	
C12	0.1165 (7)	0.5944 (6)	0.4362 (6)	0.0322 (12)	
H12	0.0745	0.5636	0.3899	0.039*	
C13	0.0393 (7)	0.7315 (7)	0.4458 (7)	0.0396 (14)	
H13	−0.0549	0.7946	0.4082	0.047*	
C14	0.1080 (8)	0.7667 (9)	0.5117 (6)	0.0467 (17)	
H14	0.0566	0.8583	0.5192	0.056*	
C15	0.2426 (8)	0.6865 (7)	0.5688 (8)	0.0437 (16)	
H15	0.2841	0.7212	0.6114	0.052*	
C16	0.3165 (8)	0.5489 (7)	0.5611 (7)	0.0427 (15)	
H16	0.4093	0.4865	0.6015	0.051*	
C17	0.3501 (6)	−0.1422 (6)	0.9394 (6)	0.0314 (12)	
C18	0.2256 (10)	−0.1633 (11)	0.9654 (7)	0.059 (2)	
H18	0.1746	−0.1651	0.9028	0.070*	
C19	0.1769 (11)	−0.1821 (12)	1.0868 (8)	0.070 (3)	
H19	0.0911	−0.1949	1.1067	0.084*	
C20	0.2564 (10)	−0.1816 (8)	1.1781 (7)	0.054 (2)	
H20	0.2231	−0.1922	1.2596	0.064*	
C21	0.3802 (12)	−0.1662 (12)	1.1503 (7)	0.068 (3)	
H21	0.4348	−0.1698	1.2136	0.081*	
C22	0.4296 (10)	−0.1447 (11)	1.0289 (7)	0.054 (2)	
H22	0.5156	−0.1323	1.0096	0.065*	
Sb2	0.0000	0.5000	1.0000	0.0698 (4)	
Cl2	−0.2299 (3)	0.5140 (4)	1.0805 (3)	0.0961 (11)	
Cl3	0.0724 (3)	0.4837 (5)	1.1918 (3)	0.1218 (17)	
Cl4	0.0999 (5)	0.2463 (5)	1.0692 (4)	0.1156 (13)	

### Atomic displacement parameters (Å^2^)

**Table:**

	*U*^11^	*U*^22^	*U*^33^	*U*^12^	*U*^13^	*U*^23^
Sb1	0.0246 (3)	0.0192 (3)	0.0262 (3)	−0.0102 (2)	0.00596 (18)	−0.01060 (19)
N1	0.031 (2)	0.020 (2)	0.029 (2)	−0.0120 (19)	0.0057 (18)	−0.0106 (17)
N2	0.030 (2)	0.021 (2)	0.028 (2)	−0.0111 (19)	0.0079 (18)	−0.0115 (17)
Cl1	0.0294 (7)	0.0389 (8)	0.0467 (8)	−0.0157 (6)	0.0003 (6)	−0.0178 (6)
C1	0.030 (3)	0.026 (3)	0.027 (2)	−0.012 (2)	0.007 (2)	−0.009 (2)
C2	0.044 (3)	0.031 (3)	0.032 (3)	−0.020 (3)	0.007 (2)	−0.007 (2)
C3	0.035 (3)	0.021 (3)	0.036 (3)	−0.011 (2)	0.001 (2)	−0.007 (2)
C4	0.027 (3)	0.023 (2)	0.031 (3)	−0.010 (2)	0.005 (2)	−0.010 (2)
C5	0.027 (3)	0.018 (2)	0.036 (3)	−0.008 (2)	0.003 (2)	−0.013 (2)
C6	0.029 (3)	0.023 (2)	0.033 (3)	−0.011 (2)	0.004 (2)	−0.013 (2)
C7	0.032 (3)	0.027 (3)	0.036 (3)	−0.010 (2)	0.011 (2)	−0.020 (2)
C8	0.039 (3)	0.034 (3)	0.033 (3)	−0.016 (3)	0.014 (2)	−0.020 (2)
C9	0.031 (3)	0.031 (3)	0.029 (3)	−0.014 (2)	0.011 (2)	−0.016 (2)
C10	0.031 (3)	0.026 (3)	0.031 (3)	−0.014 (2)	0.007 (2)	−0.012 (2)
C11	0.030 (3)	0.027 (3)	0.032 (3)	−0.014 (2)	0.008 (2)	−0.013 (2)
C12	0.028 (3)	0.025 (3)	0.042 (3)	−0.007 (2)	0.001 (2)	−0.014 (2)
C13	0.027 (3)	0.029 (3)	0.060 (4)	−0.005 (2)	0.002 (3)	−0.021 (3)
C14	0.034 (3)	0.064 (5)	0.043 (3)	−0.032 (3)	0.002 (3)	−0.003 (3)
C15	0.037 (3)	0.032 (3)	0.070 (5)	−0.012 (3)	−0.003 (3)	−0.030 (3)
C16	0.039 (3)	0.030 (3)	0.062 (4)	−0.010 (3)	−0.008 (3)	−0.021 (3)
C17	0.026 (3)	0.022 (2)	0.046 (3)	−0.008 (2)	0.003 (2)	−0.016 (2)
C18	0.061 (5)	0.096 (7)	0.046 (4)	−0.056 (5)	0.021 (4)	−0.029 (4)
C19	0.056 (5)	0.093 (7)	0.054 (5)	−0.040 (5)	0.025 (4)	−0.013 (5)
C20	0.072 (5)	0.045 (4)	0.035 (3)	−0.025 (4)	0.023 (3)	−0.012 (3)
C21	0.090 (7)	0.098 (8)	0.034 (4)	−0.055 (6)	0.015 (4)	−0.028 (4)
C22	0.055 (5)	0.088 (6)	0.038 (3)	−0.048 (5)	0.013 (3)	−0.023 (4)
Sb2	0.0346 (4)	0.1176 (8)	0.0807 (6)	−0.0245 (4)	0.0141 (4)	−0.0767 (6)
Cl2	0.0491 (13)	0.171 (3)	0.110 (2)	−0.0501 (17)	0.0332 (13)	−0.103 (2)
Cl3	0.0586 (15)	0.222 (5)	0.119 (2)	−0.039 (2)	0.0162 (15)	−0.133 (3)
Cl4	0.116 (3)	0.121 (3)	0.112 (3)	−0.034 (2)	0.039 (2)	−0.075 (2)

### Geometric parameters (Å, º)

**Table:**

Sb1—N2^i^	2.054 (4)	C11—C16	1.379 (9)
Sb1—N2	2.054 (4)	C12—C13	1.400 (8)
Sb1—N1	2.070 (4)	C12—H12	0.9400
Sb1—N1^i^	2.070 (4)	C13—C14	1.332 (10)
Sb1—Cl1^i^	2.3547 (14)	C13—H13	0.9400
Sb1—Cl1	2.3547 (14)	C14—C15	1.347 (10)
N1—C4	1.380 (7)	C14—H14	0.9400
N1—C1	1.390 (7)	C15—C16	1.396 (9)
N2—C6	1.386 (7)	C15—H15	0.9400
N2—C9	1.394 (7)	C16—H16	0.9400
C1—C10^i^	1.402 (8)	C17—C22	1.359 (10)
C1—C2	1.429 (8)	C17—C18	1.377 (10)
C2—C3	1.356 (9)	C18—C19	1.395 (11)
C2—H2	0.9400	C18—H18	0.9400
C3—C4	1.434 (8)	C19—C20	1.394 (14)
C3—H3	0.9400	C19—H19	0.9400
C4—C5	1.406 (8)	C20—C21	1.339 (13)
C5—C6	1.398 (8)	C20—H20	0.9400
C5—C11	1.508 (7)	C21—C22	1.398 (10)
C6—C7	1.430 (8)	C21—H21	0.9400
C7—C8	1.359 (9)	C22—H22	0.9400
C7—H7	0.9400	Sb2—Cl3	2.311 (3)
C8—C9	1.426 (8)	Sb2—Cl3^ii^	2.311 (3)
C8—H8	0.9400	Sb2—Cl2^ii^	2.374 (2)
C9—C10	1.393 (8)	Sb2—Cl2	2.374 (2)
C10—C1^i^	1.402 (8)	Sb2—Cl4	2.393 (4)
C10—C17	1.536 (8)	Sb2—Cl4^ii^	2.393 (4)
C11—C12	1.369 (8)		
			
N2^i^—Sb1—N2	180.00 (16)	C12—C11—C16	120.6 (6)
N2^i^—Sb1—N1	90.19 (18)	C12—C11—C5	120.1 (5)
N2—Sb1—N1	89.81 (18)	C16—C11—C5	119.1 (5)
N2^i^—Sb1—N1^i^	89.81 (18)	C11—C12—C13	120.5 (6)
N2—Sb1—N1^i^	90.19 (18)	C11—C12—H12	119.8
N1—Sb1—N1^i^	180.0 (2)	C13—C12—H12	119.8
N2^i^—Sb1—Cl1^i^	90.57 (14)	C14—C13—C12	115.6 (7)
N2—Sb1—Cl1^i^	89.43 (14)	C14—C13—H13	122.2
N1—Sb1—Cl1^i^	89.98 (14)	C12—C13—H13	122.2
N1^i^—Sb1—Cl1^i^	90.02 (14)	C13—C14—C15	127.7 (8)
N2^i^—Sb1—Cl1	89.43 (14)	C13—C14—H14	116.2
N2—Sb1—Cl1	90.57 (14)	C15—C14—H14	116.2
N1—Sb1—Cl1	90.02 (14)	C14—C15—C16	116.0 (7)
N1^i^—Sb1—Cl1	89.98 (14)	C14—C15—H15	122.0
Cl1^i^—Sb1—Cl1	180.0	C16—C15—H15	122.0
C4—N1—C1	108.0 (4)	C11—C16—C15	119.6 (6)
C4—N1—Sb1	126.0 (4)	C11—C16—H16	120.2
C1—N1—Sb1	126.0 (4)	C15—C16—H16	120.2
C6—N2—C9	108.0 (4)	C22—C17—C18	122.1 (6)
C6—N2—Sb1	126.3 (4)	C22—C17—C10	118.2 (6)
C9—N2—Sb1	125.6 (4)	C18—C17—C10	119.5 (6)
N1—C1—C10^i^	126.1 (5)	C17—C18—C19	118.5 (8)
N1—C1—C2	107.9 (5)	C17—C18—H18	120.7
C10^i^—C1—C2	126.0 (5)	C19—C18—H18	120.7
C3—C2—C1	108.1 (5)	C20—C19—C18	119.4 (8)
C3—C2—H2	125.9	C20—C19—H19	120.3
C1—C2—H2	125.9	C18—C19—H19	120.3
C2—C3—C4	107.9 (5)	C21—C20—C19	120.4 (7)
C2—C3—H3	126.0	C21—C20—H20	119.8
C4—C3—H3	126.0	C19—C20—H20	119.8
N1—C4—C5	126.6 (5)	C20—C21—C22	121.1 (8)
N1—C4—C3	108.1 (5)	C20—C21—H21	119.4
C5—C4—C3	125.4 (5)	C22—C21—H21	119.4
C6—C5—C4	124.6 (5)	C17—C22—C21	118.4 (7)
C6—C5—C11	116.2 (5)	C17—C22—H22	120.8
C4—C5—C11	119.2 (5)	C21—C22—H22	120.8
N2—C6—C5	126.6 (5)	Cl3—Sb2—Cl3^ii^	180.000 (1)
N2—C6—C7	108.1 (5)	Cl3—Sb2—Cl2^ii^	90.36 (11)
C5—C6—C7	125.3 (5)	Cl3^ii^—Sb2—Cl2^ii^	89.64 (11)
C8—C7—C6	107.7 (5)	Cl3—Sb2—Cl2	89.64 (11)
C8—C7—H7	126.2	Cl3^ii^—Sb2—Cl2	90.36 (11)
C6—C7—H7	126.2	Cl2^ii^—Sb2—Cl2	180.0
C7—C8—C9	108.7 (5)	Cl3—Sb2—Cl4	87.99 (16)
C7—C8—H8	125.7	Cl3^ii^—Sb2—Cl4	92.01 (16)
C9—C8—H8	125.7	Cl2^ii^—Sb2—Cl4	90.45 (14)
C10—C9—N2	127.0 (5)	Cl2—Sb2—Cl4	89.55 (14)
C10—C9—C8	125.5 (5)	Cl3—Sb2—Cl4^ii^	92.01 (16)
N2—C9—C8	107.5 (5)	Cl3^ii^—Sb2—Cl4^ii^	87.99 (16)
C9—C10—C1^i^	125.1 (5)	Cl2^ii^—Sb2—Cl4^ii^	89.55 (14)
C9—C10—C17	116.2 (5)	Cl2—Sb2—Cl4^ii^	90.45 (14)
C1^i^—C10—C17	118.7 (5)	Cl4—Sb2—Cl4^ii^	180.000 (1)

Symmetry codes: (i) −*x*+1, −*y*, −*z*+1; (ii) −*x*, −*y*+1, −*z*+2.

### Hydrogen-bond geometry (Å, º)

**Table:**

*D*—H···*A*	*D*—H	H···*A*	*D*···*A*	*D*—H···*A*
C7—H7···Cl3^ii^	0.94	2.76	3.490 (8)	135
C8—H8···Cl4	0.94	2.74	3.593 (8)	151
C12—H12···Cl3^iii^	0.94	2.69	3.539 (8)	151

Symmetry codes: (ii) −*x*, −*y*+1, −*z*+2; (iii) *x*, *y*, *z*−1.
